# *Trypanosoma brucei rhodesiense* infection in a Chinese traveler returning from the Serengeti National Park in Tanzania

**DOI:** 10.1186/s40249-018-0432-5

**Published:** 2018-05-21

**Authors:** Qin Liu, Xiao-Ling Chen, Mu-Xin Chen, Han-Guo Xie, Qing Liu, Zhu-Yun Chen, Yao-Ying Lin, Hua Zheng, Jia-Xu Chen, Yi Zhang, Xiao-Nong Zhou

**Affiliations:** 1 National Institute of Parasitic Diseases, Chinese Center for Disease Control and Prevention; Chinese Center for Tropical Diseases Research; WHO Collaborating Centre for Tropical Diseases; National Center for International Research on Tropical Diseases, Ministry of Science and Technology; Key Laboratory of Parasite and Vector Biology, Ministry of Health, Shanghai, 200025 China; 2 Fujian Medical University Union Hospital, Fuzhou, Fujian 350001 People’s Republic of China; 3 Fujian Provincial Center for Diseases Control and Prevention, Fuzhou, Fujian 350000 People’s Republic of China

**Keywords:** *Trypanosoma brucei rhodesiense*, Human African trypanosomiasis, Imported infection, China, Treatment, Suramin, Tanzania

## Abstract

**Background:**

Human African trypanosomiasis (HAT) is one of the most complex parasitic diseases known to humankind. It usually occurs in endemic areas in Africa, but is occasionally detected in returning travelers and migrants in non-endemic countries.

**Case presentation:**

In August 2017, a case of HAT was diagnosed in China in a traveler returning from the Masai Mara area in Kenya and the Serengeti area in Tanzania. The traveler visited Africa from 23 July to 5 August, 2017. Upon return to China, she developed a fever (on 8 August), and *Trypanosoma brucei rhodesiense* infection was confirmed by laboratory tests (on 14 August) including observation of parasites in blood films and by polymerase chain reaction. She was treated with pentamidine followed by suramin, and recovered 1 month later.

**Conclusions:**

This is the first imported rhodesiense HAT case reported in China. This case alerts clinical and public health workers to be aware of HAT in travelers, and expatriates and migrants who have visited at-risk areas in Africa.

**Electronic supplementary material:**

The online version of this article (10.1186/s40249-018-0432-5) contains supplementary material, which is available to authorized users.

## Multilingual abstracts

Please see Additional file 1 for translations of the abstract into the six official working languages of the United Nations.

## Background

Human African trypanosomiasis (HAT), also known as sleeping sickness, is caused by infection with flagellated protozoa which are transmitted by tsetse flies [1, 2]. The disease is caused by two sub-species of *Trypanosoma brucei*, namely *Trypanosoma brucei gambiense* which causes chronic disease in western and central Africa and *T. b. rhodesiense* which is associated with acute disease in eastern and southern Africa [2].

Although HAT is often clinically symptomatic, both the diagnosis and treatment of the disease are often delayed, resulting in significant mortality [3]. Moreover, management of the disease is complex and requires specific medical expertise. In general, HAT remains an uncommon disease in non-endemic countries; however the number of cases in travelers has been rising steadily in recent years [4].

Here, we report an imported case of HAT due to *T. b. rhodesiense* which was detected in a Chinese traveler who visited of Kenya and Tanzania in China in August, 2017.

## Case presentation

**Fig. 1:**
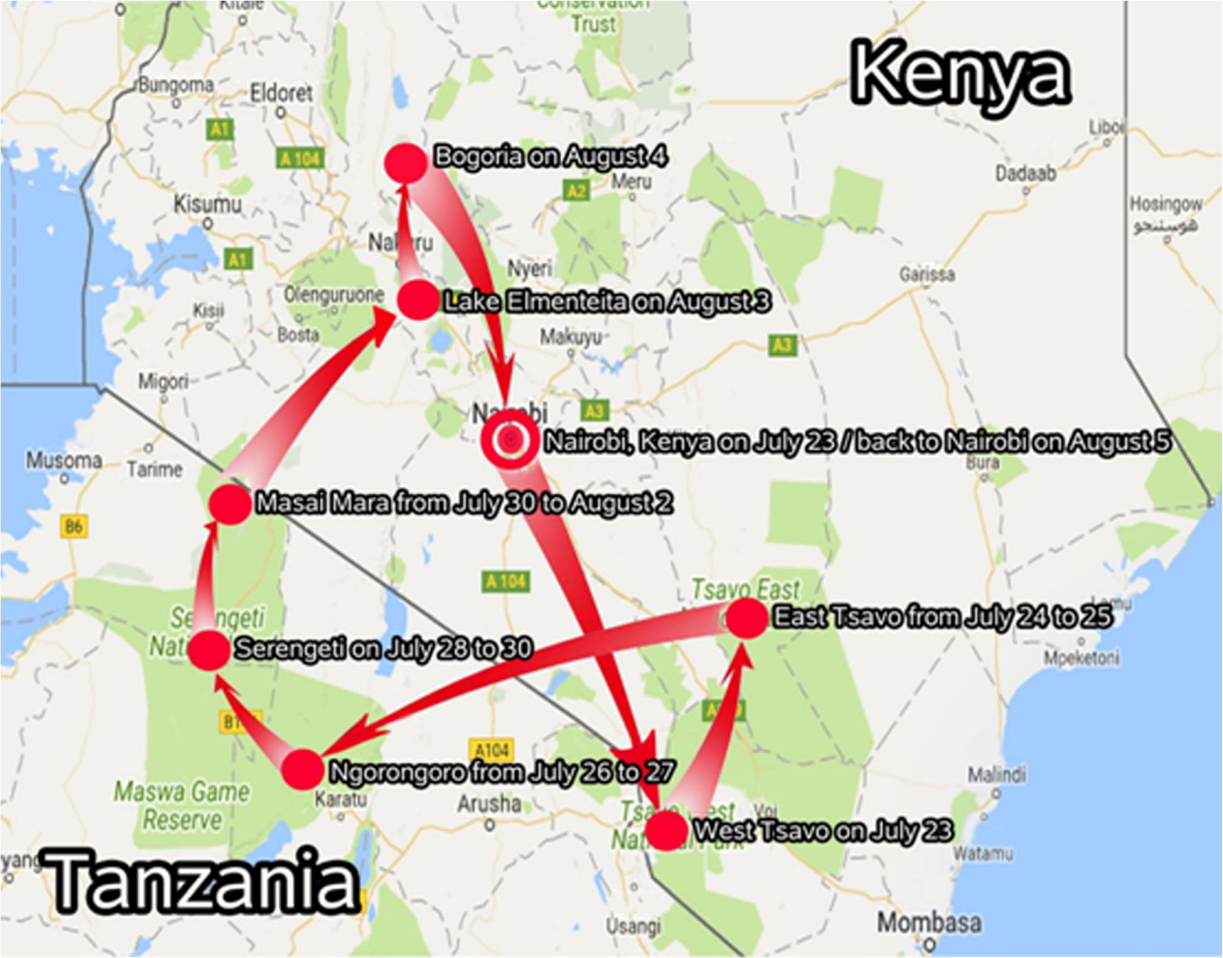
The travel route of the patient in Tanzania and Kenya

The 41-year old woman traveled to Kenya and Tanzania between 23 July and 5 August, 2017. She left China on 22 July and arrived in Nairobi, Kenya on 23 July. Her route in Kenya and Tanzania is shown in Fig. 1. She returned to China, landing at Guangzhou Airport on 6 August. Before leaving for Africa she received a yellow fever vaccination and was given advice on anti-mosquito measures to prevent malaria at a local international travel health care center.

The patient visited the Serengeti National Park in Tanzania and the Masai Mara National Reserve in Kenya between 28 July and 2 August. She was bitten by an insect identified by the local driver as a tsetse fly while taking animal photographs on 29 July at the Serengeti National Park. Her husband and the local driver were bitten by the same species of insect at the same time.

**Fig. 2:**
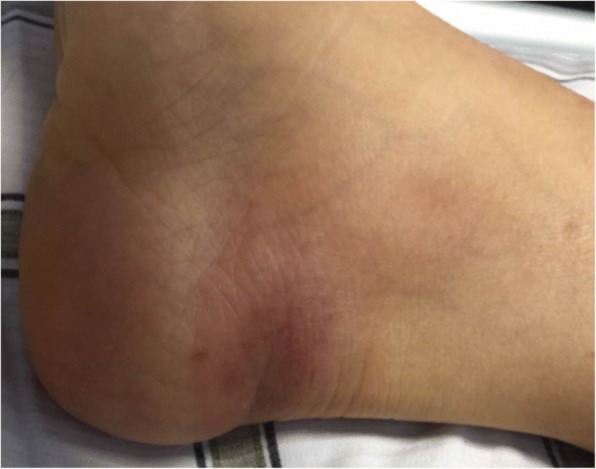
Chancre due to *Trypanosoma brucei rhodesiense* infection in a Chinese traveler returning from Serengeti, Tanzania and Masai Mara Kenya, August 2017

On 8 August, 2017, 2 days after she returned to China, she developed a fever with a temperature of 40.1 °C along with symptoms of dizziness, fatigue and rigors. She sought medical assistance at a local hospital where she was initially treated with intravenous fluids and broad-spectrum antibiotics. The following day, she was transferred to the fever department at the Fujian Medical University Union Hospital where she was examined by another physician. Clinical evaluations at the time showed that the patient was alert and well orientated, with no lymph-node enlargement or any rashes noted. The most striking abnormality was the presence of a red chancre measuring 22 mm in diameter on her right heel (see Fig. 2).

Despite the anti-infection treatment she was given, the patient remained febrile until 11 August, when she was transferred to the Infectious Diseases Department of the same hospital. At this time, her condition worsened with persistent fevers, headache, productive cough, and worsening jaundice.

Her blood tests on 14 August showed liver dysfunction (glutamic pyruvic transaminase [ALT]: 212.0 IU/L [0–40 IU/L], glutamic-oxaloacetic transaminase [AST]: 168.0 IU/L [0–46 IU/L], alkaline phosphatase: 460.0 IU/L [3–104 IU/L]). In addition she was noted to have hyponatremia (131.2 mmol/L [135.0–148.0 mmol/L]), hypokalemia (3.11 [3.5–5.5 mmol/L]), and thrombocytopenia (70 × 10^9^/L [[100–300] × 10^9^/L]). A computed tomography (CT) scan of her chest showed a strip shadow in both lungs, which the doctor thought could be pneumonia.

**Fig. 3:**
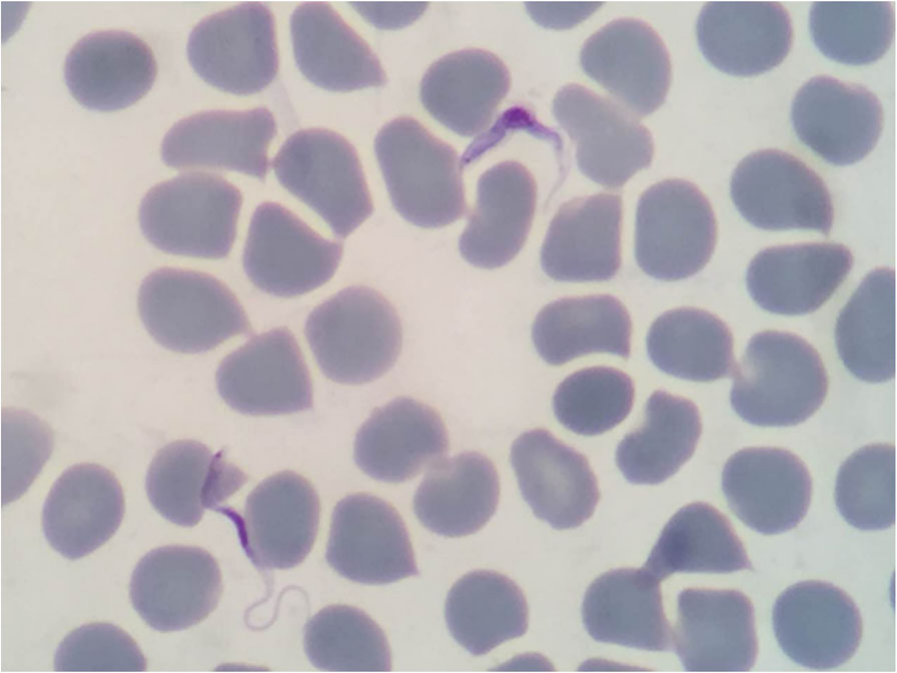
*Trypanosoma* species confirmed in a Giemsa-stained thin blood film from a Chinese traveler returning from Serengeti, Tanzania and Masai Mara Kenya, August 2017


On 14 August, the patient’s blood sample was sent to the Fujian Provincial Center for Disease Control and Prevention to investigate the presence of *Plasmodium* species. The giemsa-stained thin and thick blood smears as well as the malaria antigen test (immuno chromatographic test) were both negative for the *Plasmodium* species. However, a few trypanosomes were found in both the thin and thick blood smears. Blood examination confirmed a high parasitemia, with one to two trypanosomes seen every five fields under microscopy at 1000× magnification (see Fig. 3 and Additional file 2: Video 1).

**Additional file 2:** Video 1 Live trypanosome in wet blood film under 1000× magnification. (MP4 2775 kb)


As the presence of trypanosomes in the blood was confirmed, a normal cerebrospinal fluid (CSF) puncture performed on the same day revealed no trypanosome with two white blood cells (WBCs)/mm^3^, which signified first-stage disease.

To confirm the diagnosis of rhodesiense HAT on a molecular level, polymerase chain reaction (PCR) techniques were used. Nucleic acid DNA was extracted from the patient’s peripheral blood using the DNeasy® Blood & Tissue Kit (QIAGEN, Germany). Two specific genes namely *T. b. rhodesiense*-specific human serum resistance--associated (SRA) gene and the *Trypanosoma* spp. universal internal transcribed spacer (ITS) gene, were targeted by the PCR method, as previously described [5, 6]. After PCR products were sequenced both for SRA and ITS genes, it was found that 284 bp of the SRA gene sequence obtained match 100% to the *T. b. rhodesiense* partial SRA genes (GenBank accession numbers: Z37159, AJ345058 and AJ345057), and 450 bp of the ITS gene sequence was 97% similar to *T. brucei* isolate ITS gene (GenBank accession numbers: JX910373 and AF306771).

Since no suramin was available immediately after diagnosis, 200 mg pentamidine was initially given by intravenous (IV) injection on 15 August, and the same dose was then injected intramuscularly on 16 August and 17 August. On 16 August, after the second dose of pentamidine, no trypomastigotes were detected in thin and thick blood films. After three doses of pentamidine, the patient’s condition rapidly improved, her fever disappeared, the chancre reduced in size and the cough lessened, although her headache persisted. The liver function tests and electrolytes improved, but the platelet counts increased rapidly, reaching a level of 374 × 10^9^/L ([100–300] × 10^9^/L).

Suramin was available on 18 August provided by the World Health Organization (WHO). Pentamidine was discontinued and suramin was given at a test dose of 200 mg by IV injection on 18 August, and the treatment dose was then escalated to 1 g on days 3, 7, 14, 21 and 28. No adverse reactions were observed during treatment. After three doses of 1 g suramin, the patient’s liver function indices and electrolytes normalized. The platelet index, however, continued to rise, peaking at 588 × 10^9^/L after the second 1 g dose of suramin, but with the third dose, the platelet index began to decrease (434 × 10^9^/L). At this time, the patient felt much better, but continued to have a mild headache and cough. A new CSF analysis performed on day 13 (30 August) post-suramin initiation revealed an absence of trypanosomes and 2 WBC/mm^3^.

The patient was asymptomatic except for an occasional headache 1 month after the suramin treatment commenced. All blood test alterations had normalized at this point.

## Discussion and conclusions

A literature search conducted using PubMed revealed that 85 rhodesiense HAT cases were reported in non-endemic countries from 2000 to 2017 [7–14]. The vast majority of these cases occurred in Europe and North America, with only one suspected rhodesiense HAT infection reported in Asia [7]. This report details the first ever rhodesiense HAT case to be diagnosed in China. It is the second HAT case to be described in China, following a gambiense HAT infection reported in 2014 [15].

Most reported rhodesiense HAT cases have been from the Serengeti National Park in Tanzania, which is in direct vicinity to Masai Mara of Kenya [7, 11–14]. This patient confirmed she was bitten by a tsetse fly in Serengeti, which is likely to be the site of exposure. This finding should alert clinics and public health workers to at least include this infection in their list of differentials, especially in travelers who have visited the aforementioned endemic areas in Africa. It also underlines the importance of advising travelers to take protection measures to avoid tsetse fly bites whist visiting at-risk areas. This case has been reported to the WHO in Geneva, Switzerland, thereby providing valuable information to help control and monitor the disease, as well as highlighting risk areas for tourists.

Rhodesiense HAT is rarely seen in non-endemic countries. Recent descriptions of the rhodesiense HAT symptomatology of travelers are markedly different from the usual descriptions of African patients [16]. Therefore, it is essential that diagnostic capacities are developed in these countries since history and clinical symptoms are not always typical. For instance, in this case: (i) the clinical symptoms were not all typical of rhodesiense HAT, while it was acute febrile with headache and cough, lymph node enlargements and generalized rashes were absent during the entire disease course; (ii) the patient developed a characteristic chancre at the site of the tsetse fly bite. This clinical sign is very specific and therefore helpful in alerting clinicians to suspect rhodesiense HAT infection in returning travelers; (iii) the time period between the tsetse bite (29 July) and the onset of the clinical symptoms (8 August) was 10 days, which is consistent with the latent period of rhodesiense HAT that varies between one to 3 weeks [1], and (iv) more importantly, trypanosomes were easily detected in thick or thin blood films of the patient, indicating early high parasitemia, which is differents from what happens in patient with gambiense HAT. In this case, the high parasitemia of one or two trypanosomes detected at every five fields of the microscopy at 1000× magnification and the aforementioned signs all pointed to rhodesiense HAT. However, if the suspicion with epidemiology history and symptom is high, it is important to repeat the thick film in those which the first blood smear is negative, in order for the infection not to be missed [17].

If molecular techniques are available, it is important to confirm the sub-species of HAT that caused the infection. Because there is a wide diversity of clinical presentations between the different types of HAT, and treatments may be different. In this patient, we found that the PCR method was efficient in detecting the relevant nucleic acid of the parasite. Several primers including SRA B537/SRA B538, SRA651/SRA652, SRA-F1/SRA-R1 and ITS genes were used for the PCR [5, 6, 18, 19]. Results from the PCR showed that SRA-F1/SRA-R1 and ITS primers had higher sensibility and specificity. In general, SRA-F1/SRA-R1 primers were easier to amplify and thus to identify *T. b. rhodesiense* in this case report and are recommended for the diagnosis of imported cases of the parasitic disease in non-endemic areas.

In the laboratory examinations, most biochemical indexes were normal except the liver function indices and levels of Na^+^, K^+^ and platelets counts in the blood. The total bilirubin, ALT, AST, and alkaline phosphatase increased and Na^+^, K^+^ and platelet counts decreased at the point of diagnosis, with all of these normalizing after treatment with pentamidine and suramin. This finding has been previously reported [20].

In terms of haematological indices, the platelet count was low before treatment, it rose rapidly after drugs were administered, and decreased gradually in the later phase. This is consistent with reports that HAT may cause relative thrombocytopenia [20]. It has been postulated that the heat labile protein of *T. b. rhodesiense* has direct toxic effect on platelets leading to a decline in numbers [21]. Rebound thrombocytosis could be related to the rapid maturation of large numbers of bone marrow megakaryocytes and the release of platelets into the circulation following chemotherapy [20]. In general, liver function indices, Na^+^, K^+^ and platelets act as helpful diagnostic and monitoring clues in *T. b. rhodesiense* infection. However, alterations in these indices are non-specific, and may occur in other imported infections such as malaria, typhoid fever, and rickettsial disease, which may be different in specific circumstances.

The earlier a HAT infection is treated, the better the sequalae are in terms of tolerability and overall-cure rate [1]. This case highlights a particular challenge for access to treatment for rare but serious imported infections in non-endemic countries [22]. All five routine treatment drugs for HAT are donated by the manufacturers to WHO headquarters in Geneva, and can be delivered free of charge by the WHO to non-endemic countries when a case in identified. However, delivery of these drugs can take time, and there may be logistical challenges related to their import. In this patient, before the arrival of suramin from the WHO, the second-line drug, pentamidine, was obtained from Hong Kong of China. It is be ideal that China retains a stock of these drugs to treat future rare imported cases without any delay. Therefore, this case could provide a stimulus to rapid access of these essential drugs through the mechanism of drug storage at regional level, along with capacity-building in diagnosis and treatment-expertise at the national level.

Follow-up after treatment is an essential part of any HAT management strategy. Although relapses after treatment with first-stage drugs such as pentamidine and suramin are rare, drug resistance of *T. b. rhodesiense* isolates from Tanzania have been reported [23]. Therefore, it was suggested to the patient to follow up at the first, third, sixth and 12th months. This will involve clinical, blood tests and a CSF check [21, 24]. In addition, the patient was advised to consult her physician should clinical symptoms reappear.

As demonstrated in this case, the patient acquired rhodesiense HAT through the classical method of exposure: as a tourist visiting game reserves in east and south of Africa [25]. Therefore pre-travel health education is necessary for all visitors planning to visit these endemic areas. This includes providing information on the precautions to take against tsetse bites by avoiding specific places known as tsetse habitats and, if possible, wearing long sleeves and pants, and not wearing clothes in dark colours (especially blue and black) [1, 22].

This paper describes a classic case of rhodesiense HAT in a non-endemic country. Rhodesiense HAT currently occupies only 3% of the total global HAT burden, however it is more commonly seen than gambiense HAT in non-endemic countries [4, 26]. Although there has been a substantial decline in total HAT infections worldwide, thanks to a concerted WHO elimination campaign, the number of cases of rhodesiense HAT has remained stable in recent years probably due to challenges in dealing with the animal reservoir [26]. It is likely there will be reports of cases of both types of HAT in non-endemic countries in the future.

This is the first imported rhodesiense HAT case reported in China. With increased population exchanges between China and Africa in our globalized world, greater possibilities will occur for these diseases to spread. Rapid diagnosis and proper treatment are crucial to the sequelae of HAT patients. This case should alert clinical and public health workers to be aware of HAT in travlers and migrants who have visited at-risk areas in Africa.

## Additional files

Additional file 1: Multilingual abstracts in the six official working languages of the United Nations. (PDF 825 kb)

## Abbreviations

ALT: Glutamic pyruvic transaminase
AST: Glutamic-oxalacetic Transaminase
CSF: Cerebrospinal fluid
CT: Computed tomography
HAT: Human African Trypanosomiasis
ITS: Internal transcribed spacer gene
IV: Intravenous
PCR: Polymerase chain reaction
SRA: Specific human serum resistance associated gene
WBC: White blood cells
WHO: World Health Organization
