# Supplementary material for: Trypanosoma brucei rhodesiense infection in a Chinese traveler returning from the Serengeti National Park in Tanzania
Source: Infect Dis Poverty. 2018 May 21;7:50. doi: 10.1186/s40249-018-0432-5 (PMC5961482; doi:10.1186/s40249-018-0432-5)
Supplement: Supplementary file 2 — Multilingual abstracts in the six official working languages of the United Nations. (PDF 825 kb) [file 40249_2018_432_MOESM1_ESM.pdf]

Translation of the abstract into the six official working languages of the United Nations

إصابة مسافرة صينية بعدوى المثقبية البروسية الروديسية بعد عودتها من متنزه سيرنجتي الوطني بتنزانيا

Qin Liu, Mu-Xin Chen, Xiao-Ling Chen, Han-Guo Xie, Qing Liu, Zhu-Yun Chen, Yao-Ying Chen, Hua Zheng, Jia-Xu Chen, Yi Zhang, Xiao-Nong Zhou

الملخص :

خلفية : يعد داء المثقبيات الأفريقي البشري واحدا من أكثر الأمراض الطفيلية التي عرفتها البشرية تعقيدا.
وتكثر الإصابة به في المناطق االموطونة بأفريقيا , لكن يتم رصد العدوى بين الحين والآخر في المناطق الغير موطونة نتيجة إصابة بعض المسافرين العائدين أو المهاجرين بالعدوى

عرض الحالة : في أغسطس عام 2017 , تم رصد حالة مصابة بداء المثقبيات الأفريقي البشري في الصين بعد عودة مسافرة كانت في زيارة لمنطقة ماساي مارا في كينيا ومتنزه سيرنجتي في تنزانيا
حيث كانت المسافرة في زيارة لأفريقيا في الفترة ما بين الثالث والعشرين من شهر يوليو وحتى الخامس من أغسطس ,2017.
وبعد وصولها إلى الصين أصيبت بالحمى (تحديدا في الثامن من أغسطس) , و أكدت نتائج الاختبارات المعملية في الرابع عشر من أغسطس إصابتها بعدوى المثقبية البروسية الروديسية بعد أن وجدت الطفيليات في فلم الدم وأجري تحليل تفاعل البلمرة التسلسلي.
وعولجت المريضة عن طريق تناول عقار البنتاميدين متبوعا بعقار السورامين وتعافت بعد مرور شهر.

الاستنتاجات : هذه هي أول حالة وافدة مصابة بداء المثقبيات الأفريقي البشري الروديسي يتم رصدها في الصين.
ويعد ذلك تنبيها للأطباء والعاملين في مجال الصحة العامة ليصبحوا أكثر إدراكا لإحتمالية إصابة المسافرين بداء المثقبيات الأفريقي البشري خاصة العائدين منهم من المناطق المعرضة لخطر انتشار العدوى بأفريقيا.

Translated from English version into Arabic by Salma Adel, proofread by Tahir Abba, through

TRANSLATORS
WITHOUT BORDERS

一例输入性的布氏罗德西亚锥虫病病例报道

刘琴，陈木新，陈晓玲，谢汉国，刘青，陈朱云，林耀莹，郑华、陈家旭、张仪，周晓农

摘要

引言：人非洲锥虫病（Human African trypanosomiasis ，HAT）是一种复杂的寄生虫病。它通常发生在非洲的流行区，但偶尔也发生于非流行国家的从疫区归来的旅游者和移民。

病例介绍：2017 年 8 月，一位从非洲肯尼亚和坦桑尼亚归来的中国的游客被诊断为非洲锥虫病。这名游客于 2017 年 7 月 23 日至 2017 年 8 月 5 日游玩了非洲肯尼亚马赛玛拉地区和坦桑尼亚的塞伦盖蒂地区。在回到中国后，她于两天后（8 月 8 日）开始发烧，于 8 月 14 日经实验室寄生虫血涂片镜检和 PCR 证实感染布氏罗得西亚锥虫。随后，她经过戊脒烷和苏拉明药物治疗，一个月后康复。

结论：这是中国报道的第一例输入性的布氏罗得西亚锥虫病感染病例。这一病例提醒临床医生和公共卫生工作人员留意访问过非洲高危地区的旅行者、外籍人士以及移民这些人群感染非洲锥虫病的可能。

Translated from English version into Chinese by Qin Liu

Infestation par *Trypanosoma brucei rhodesiense* chez une voyageuse chinoise revenant du parc national du Serengeti en Tanzanie

Qin Liu, Chen Xin-Chen, Xiao-Ling Chen, Xie-Li Xie, Liu Qing, Zhu-Yun Chen, Chen Yao-Ying, Hua Zheng, Jia-Xu Chen, Yi Zhang, Xiao-Nong Zhou

Résumé

Contexte: La trypanosomiase humaine africaine (THA) est l'une des maladies parasitaires les plus complexes que l'on connaisse. Elle survient généralement dans les zones d'endémie en Afrique, mais elle est parfois détectée chez les voyageurs de retour et les migrants dans les pays non endémiques.

Présentation du cas: En août 2017, un cas de THA a été diagnostiqué en Chine chez une voyageuse revenant de la région de Masai Mara au Kenya et de la région de Serengeti en Tanzanie. Cette voyageuse s'est rendue en Afrique du 23 juillet au 5 août 2017. À son retour en Chine, elle a développé de la fièvre (le 8 août) et une infection par *Trypanosoma brucei rhodesiense* a été confirmée par des tests de laboratoire (le 14 août) incluant l'observation de parasites dans les frottis sanguins et une réaction en chaîne à la polymérase. La patiente a été traitée avec de la pentamidine puis de la suramine et déclarée guérie un mois plus tard.

Conclusions: Il s'agit du premier cas de THA à *T. rhodesiense* importé signalé en Chine. Ce cas avertit les agents de santé publique et clinique de la possibilité que les voyageurs, ainsi que les expatriés et les migrants qui ont visité des zones à risque en Afrique, reviennent porteurs d'une THA.

Translated from English version into French by Abdourahamane, proofread by Appen 5, through

TRANSLATORS WITHOUT BORDERS

У путешественника из Китая после возвращения из национального парка Серенгети в Танзании выявили **заражение trypanosoma brucei rhodesiense**

Цинь-Лю, Му-Синь Чэнь, Сяо-Лин Чэнь, Хан-Го-Се, Цин Лю, Чжу-Юнь Чэнь, Яо-Ин Чэнь, Хуа Чжэн, Цзя-Сюй Чэнь, Йи Чжан, Сяо-Нон Чжоу

Аннотация

Исходные данные: Африканский трипаносомоз человека (НАТ) является одним из наиболее сложных паразитарных заболеваний в истории человечества. Как правило, оно возникает в эндемических районах Африки, но иногда его выявляют у туристов и мигрантов, возвращающихся в неэндемические страны.

Презентация случая: В августе 2017 года у китайской туристки, которая вернулась из районов Масаи-Мара в Кении и Серенгети в Танзании обнаружили заражение НАТ. Путешественницв находилась в Африке с 23 июля по 5 августа 2017 года. Сразу после возвращение в Китай у неё началась лихорадка (8 августа), и лабораторные тесты, а также наблюдение за паразитами в мазках крови и цепная реакция полимеразы, подтвердили (14 августа), что это следствие распространения *инфекции* trypanosoma brucei rhodesiense. Ей был прописан сначала пентамидин, затем сурамин, и спустя месяц она выздоровела.

Выводы: Это первый зарегистрированный случай заражения HAT в Китае, завезённый из другой страны. Этот случай предупреждает персонал клинической практики и здравоохранения о присутствии риска заражения HAT со стороны путешественников, экспатриантов и мигрантов, которые возвращаются из районов повышенного риска Африки.

Translated from English version into Russian by Kate Kazyk, proofread by Liudmila Tomanek, through

TRANSLATORS
WITHOUT BORDERS

Infección de *Trypanosoma brucei rhodesiense* en una viajera china que regresaba del Parque Nacional Serengueti de Tanzania

Qin Liu, Mu-Xin Chen, Xiao-Ling Chen, Han-Guo Xie, Qing Liu, Zhu-Yun Chen, Yao-Ying Chen, Hua Zheng, Jia-Xu Chen, Yi Zhang, Xiao-Nong Zhou

Resumen

Antecedentes: la tripanosomiasis humana africana (HAT, por sus siglas en inglés) es una de las enfermedades parasitarias más complejas que conocemos. Usualmente se detecta en zonas endémicas de África, aunque también ocasionalmente en países no endémicos en viajeros y migrantes que regresan.
Presentación del caso: en agosto de 2017, se diagnosticó un caso de HAT en China, en una viajera que regresaba del área de Masái Mara en Kenya y el área de Serengueti en Tanzania. La viajera visitó África del 23 de julio al 5 de agosto de 2017. Tras su regreso a China, desarrolló fiebre (el 8 de agosto) y se confirmó que estaba infectada de *Trypanosoma brucei rhodesiense* por medio de análisis de laboratorio (el 14 de agosto) en que se observaron parásitos en extensión sanguínea y por reacción en cadena de la polimerasa. Se la trató con pentamidina seguida de suramina, y se recuperó luego de un mes.
Conclusiones: se trata del primer caso importado de HAT rhodesiense del que se tiene conocimiento en China. Este caso advierte a los trabajadores de salud pública y clínica sobre la posibilidad de que los viajeros y expatriados y migrantes que hayan visitado áreas de riesgo en África tengan esta enfermedad.

Translated from English version into Spanish by Macarena Belén Pierrot, proofread by Maria Esther Fernandez, through

TRANSLATORS
WITHOUT BORDERS
